# Feasibility of force myography for the direct control of an assistive robotic hand orthosis in non-impaired individuals

**DOI:** 10.1186/s12984-023-01222-8

**Published:** 2023-08-03

**Authors:** Jessica Gantenbein, Chakaveh Ahmadizadeh, Oliver Heeb, Olivier Lambercy, Carlo Menon

**Affiliations:** 1 Rehabilitation Engineering Laboratory, Department of Health Sciences and Technology, ETH Zurich, Lengghalde 5, 8008 Zurich, Switzerland; 2 Biomedical and Mobile Health Technology Lab, Department of Health Sciences and Technology, ETH Zurich, Lengghalde 5, 8008 Zurich, Switzerland; 3 Future Health Technologies, Singapore-ETH Centre, Campus for Research Excellence And Technological Enterprise (CREATE), 1 Create Way, Singapore, 138602 Singapore

**Keywords:** Force myography, Robotic hand orthosis, Gesture recognition, Human–machine interfaces, Intention detection

## Abstract

**Background:**

Assistive robotic hand orthoses can support people with sensorimotor hand impairment in many activities of daily living and therefore help to regain independence. However, in order for the users to fully benefit from the functionalities of such devices, a safe and reliable way to detect their movement intention for device control is crucial. Gesture recognition based on force myography measuring volumetric changes in the muscles during contraction has been previously shown to be a viable and easy to implement strategy to control hand prostheses. Whether this approach could be efficiently applied to intuitively control an assistive robotic hand orthosis remains to be investigated.

**Methods:**

In this work, we assessed the feasibility of using force myography measured from the forearm to control a robotic hand orthosis worn on the hand ipsilateral to the measurement site. In ten neurologically-intact participants wearing a robotic hand orthosis, we collected data for four gestures trained in nine arm configurations, i.e., seven static positions and two dynamic movements, corresponding to typical activities of daily living conditions. In an offline analysis, we determined classification accuracies for two binary classifiers (one for opening and one for closing) and further assessed the impact of individual training arm configurations on the overall performance.

**Results:**

We achieved an overall classification accuracy of 92.9% (averaged over two binary classifiers, individual accuracies 95.5% and 90.3%, respectively) but found a large variation in performance between participants, ranging from 75.4 up to 100%. Averaged inference times per sample were measured below 0.15 ms. Further, we found that the number of training arm configurations could be reduced from nine to six without notably decreasing classification performance.

**Conclusion:**

The results of this work support the general feasibility of using force myography as an intuitive intention detection strategy for a robotic hand orthosis. Further, the findings also generated valuable insights into challenges and potential ways to overcome them in view of applying such technologies for assisting people with sensorimotor hand impairment during activities of daily living.

## Background

Neurological injuries, such as spinal cord injury or stroke, can lead to impaired hand function and thus may have a significant impact on the independence and quality of life of the affected individuals. Assistive devices, such as robotic hand orthoses (RHO), can help their users regain independence by supporting grasping function during everyday tasks [1]. However, reliably and safely detecting their movement intention for device control is crucial to gain the full benefit from using such devices.

For many RHO used commercially or in research, conventional direct control inputs, such as buttons or joysticks, are used to control the actions of the device [2, 3]. These inputs are often the strategy of choice due to their robustness and ease-of-use. However, operating such inputs is detached from the physiological movement generation and thus is presumably neither perceived as natural by their users, nor is expected to promote neuroplasticity and consequently motor recovery. To alleviate this issue, strategies based on non-invasive measurements of biosignals provide options for a potentially more natural control.

The most conventional biosignal used for the control of assistive devices is surface electromyography (sEMG), and has shown its potential for gesture recognition in both impaired and unimpaired populations [4–6]. However, the quality of a sEMG signal is sensitive to sources of noise from the surroundings or the used hardware, sweat, signal crosstalk and electrode shifts, making its prolonged use as an intention detection strategy challenging [5]. Force myography (FMG), i.e., monitoring interface forces between the limb and a wearable such as, e.g., an instrumented textile band, resulting from volumentric changes in the muscles during contraction and relaxation, poses a promising alternative [7]. Multiple studies have shown that FMG can perform comparably to sEMG in terms of classification accuracy in gesture recognition tasks [8, 9] or even reported that FMG outperformed EMG in lower muscle activation levels [10]. Furthermore, other works have shown FMG to be more stable over time [11, 12] and more robust to concurrent activation by undesired actions [10]. This is specifically relevant in the context of assistive RHO considering their target population, i.e., people with weakness and reduced muscular activity in their upper limbs. Despite these promising indications, the studies using FMG to control upper-limb assistive devices are still relatively scarce. Primarily, FMG has been used in gesture classification for prosthetics [7, 8, 13, 14]. Only a limited number of studies have also exploited FMG for RHO control, whereas in most cases the signal was collected from the forearm contralateral to the worn RHO [15, 16]. However, measuring FMG from the contralateral side restricts the conduction of bimanual tasks which are crucial for many activities in daily living. Thus, for RHO which are primarily targeting assistive applications in activities of daily living, contralateral control is not fully representative. Only a few studies collected FMG signals from the forearm ipsilateral to the RHO [17, 18], yet did not evaluate this strategy quantitatively.

In this work, we explored the feasibility of using FMG signals collected from the forearm using a custom-made wearable to distinguish different grasp patterns and intuitively control an assistive RHO worn on the hand ipsilateral to the wearable in an offline manner. Further, in view of a real-world application, we attempted to optimize classifier training procedures in terms of time and complexity by determining the training arm configurations that achieved the highest classificaton accuracies. The analysis of the data collected from neurologically-intact participants encouraged the further implementation of such a technique for RHO control, but also underlined the additional challenges which may arise when transitioning to application for people with neurological impairments.

## Materials and methods

### FMG band

**Fig. 1:**
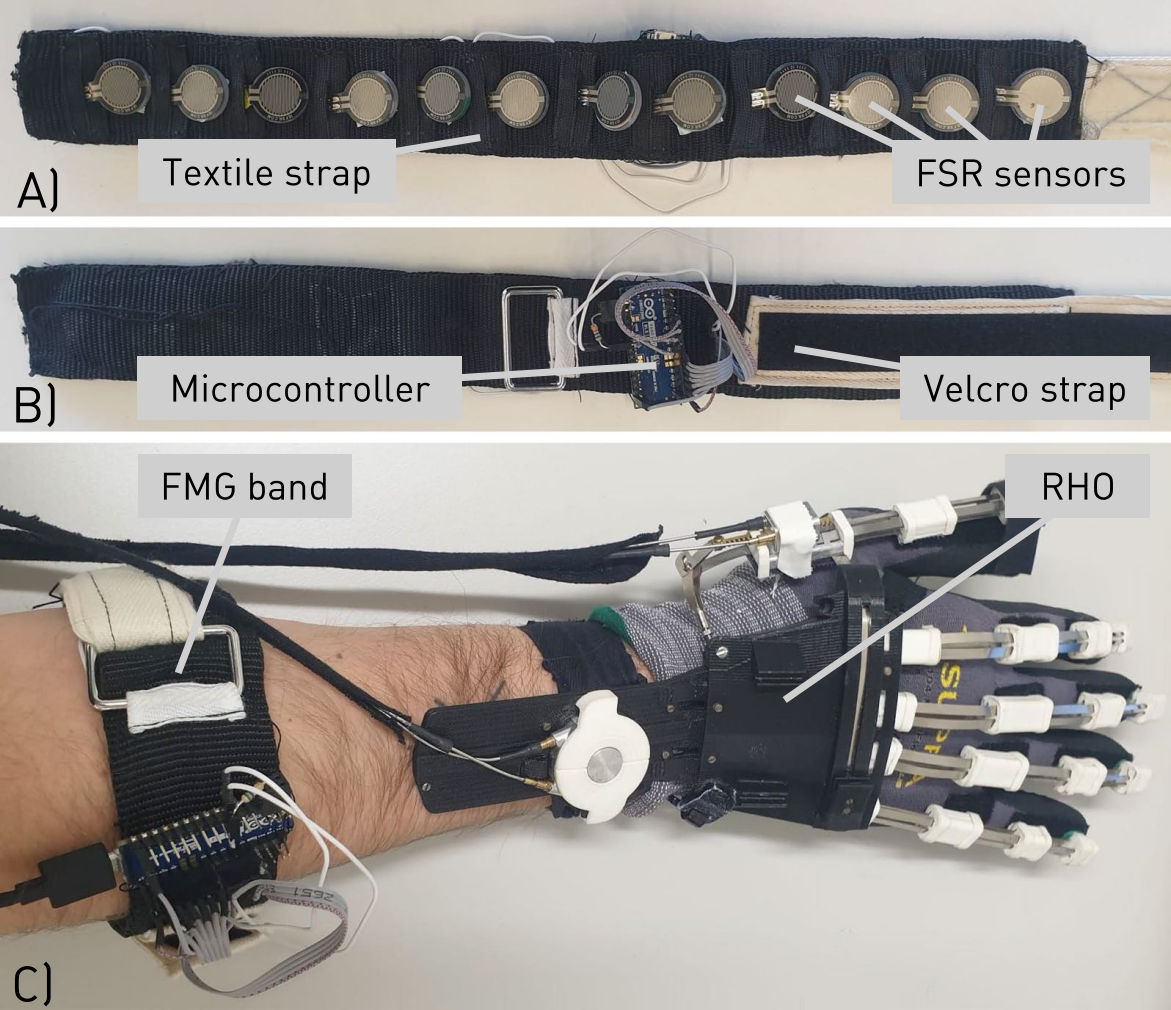
Overview of the hardware setup, i.e. FMG band and RHO: **A** Inner layer of the custom-built FMG band consisting of a textile strap with 12 FSR sensors incorporated. **B** Outer layer of the custom-built FMG band consisting of a textile strap with an incorporated microcontroller for signal acquisition and a Velcro strap to allow for adjustable sizing. **C** FMG band worn on the forearm and RHO donned

Using an instrumented armband has shown to be a valid approach to detect relevant changes in FMG signals [19, 20] and allows for simplified sensor placement. Due to the lack of suitable commercially available solutions, a custom-built sensor band similar to the one used used by Xiao and Menon [19] was designed (Fig. 1A, B). To detect the force generated by the muscle’s volume changes in the forearm during muscle activations, force-sensitive resistors (FSRs) were used. Twelve FSRs (FSR402, Interlink Electronics, Inc., Los Angeles, CA) were embedded on the inner textile layer of the band with a 3cm distance between two consecutive sensors. The resulting band was suitable for arm circumferences between 20cm and 36cm which covers adult forearm circumferences reported by Delva et al. [21]. Circular acrylic plates were fixed on the back of the sensing area of the FSRs to enable a more even pressure distribution, and increase sensitivity for a consistent sensor read-out [22].

Signal collection and analog-digital-conversion was done on a microcontroller attached to the band (Arduino Nano BLE, Arduino s.r.l., Monza, Italy), where each FSR was connected to an individual digital output and a shared analog input. To quantify individual sensor readings at the analog input, a voltage divider circuit was implemented using a 56 kOhm pull-down resistor [17]. As commonly applied for FMG data acquisition [23], the targeted sampling frequency was approximately 100 Hz. The collected data was sent via USB to a computer running a Python script where it was saved for further analysis.

### Robotic hand orthosis

In this study, we investigated whether FMG signals could be used to control a fully wearable and portable RHO, the RELab tenoexo (Fig. 1C). This RHO has been designed to support grasp function in people with sensorimotor hand impairments through active assistance in finger flexion and extension [24]. Its fingers consist of a three-layered sliding spring mechanism, which allows for a compliant grasping of objects of different sizes and shapes without the need to control individual interphalangeal joints [25]. Two Bowden cable-based remote actuation systems transmit the force from the motors stored in a back module to the spring mechanism in the hand module [26]. One system actuates thumb flexion and extension, while the second actuates combined flexion and extension of the remaining four fingers. A manual slider allows for thumb opposition to enable the most relevant grasp types for daily tasks.

### Recruitment

Participants were recruited at ETH Zurich for a single experimental session. All participants gave written informed consent, and all experimental procedures were approved by the ethics committee of ETH Zurich (2021-N-171). Neurologically-intact individuals with no impaired hand function who were above 18 years of age, able to give informed consent and understand the tasks involved in the study were eligible to participate. Major depression or deficits in cognition, communication, comprehension, or memory were defined as exclusion criteria.

A total of ten participants were enrolled in the study (all male, mean age 27.2 years, standard deviation *SD* = 2.2 years). Forearm circumferences ranged between 26 and 30 cm (mean: 27.0 cm, *SD* = 1.6 cm).

### Experimental protocol

**Fig. 2:**
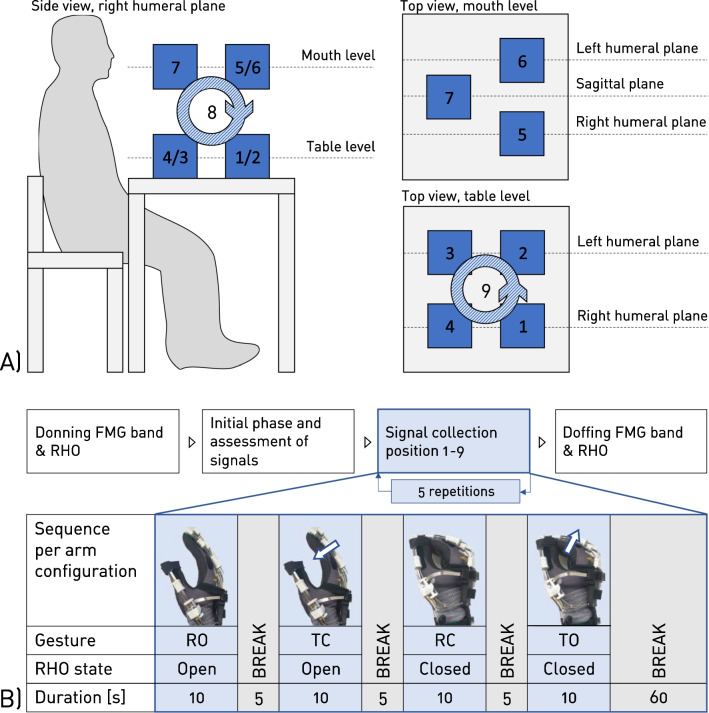
Study setup and protocol: **A** Study setup depicting the participant sitting in front of a table and the seven static positions (1–7) and two dynamic movements (8–9) assessed in the protocol. **B** Study protocol consisting of donning, an initial phase, the signal collection phase of five repetitions per gesture and arm configuration, and donning

The full study setup and protocol is depicted in Fig. 2. Participants were seated at a table and had the RHO donned on their right hands. The FMG band was donned on the ipsilateral (i.e., right) forearm at the location of the largest circumference and rotated until the microcontroller was placed above the brachioradialis muscle. The band was adjusted until it was tight, yet comfortable as subjectively reported by the participants. Depending on the participant’s forearm circumference, only signals from sensors needed for one full loop around the forearm were acquired and sensors laying on top of those were considered inactive, resulting in a lower number of active sensors. The state of the RHO, i.e., “open” and “closed”, was manually changed by the examiner using a push button for the duration of the full experiment.

In an initial phase, the participants were instructed to try performing four gestures while wearing the RHO. While the state of the RHO was open, the two gestures were “relaxed open” (*RO*) and “try to close” (*TC*). For the gesture *TC*, the participants had to try to close their hand while the closing movement was counteracted by the force generated by the finger spring mechanism of the RHO. Vice versa, when the state of the RHO was closed, the two gestures were “relaxed closed” (*RC*) and “try to open” (*TO*). Participants were instructed to perform the two “not relaxed” gestures *TO* and *TC* with approximately half of their individually perceived maximal force. During this initial phase, a first visual inspection of signal quality was performed before the commencement of the experiments.

**Fig. 3:**
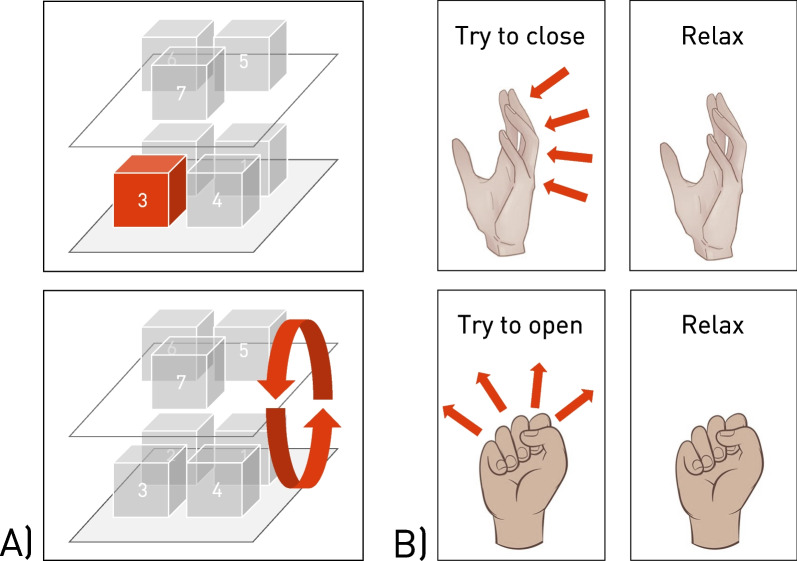
Visual cues provided to the participants **A** Visuals cueing the current arm configuration to the participants, examples shown for static position 3 (top) and dynamic movement 8 (bottom). **B** Visuals cueing one of the four gestures to be performed to the participants

In the data collection phase, participants were asked to perform one of the four gestures while in a specific arm configuration. A sequence of performing each gesture continuously for 10s followed by a 5s break was conducted (Fig. 2B) and repeated for a total of nine arm configurations, i.e., seven static hand positions (positions 1–7) and two dynamic movements (movement 8–9) with a break of 60s after each arm configuration as depicted in Fig. 2A. The static positions were selected based on the positions proposed by Radmand et al. [27] and their relevance in terms of activities of daily living that could be supported by the RHO. Since the intended target population of the RHO, i.e., people after spinal cord injury with upper limb weakness, predominantly are wheelchair users, the proposed positions below table level and above head level were left out. The two closer positions at face level were fused into a single position in front of the participants’ mouth, since eating and drinking have been reported to be among the most desired tasks for target users of RHO [28]. The two dynamic movements consisted of a circular movement horizontally on table level and vertically in the right humeral plane, respectively. For all arm configurations and all gestures, the participants were instructed to sit comfortably and relaxed, and to have their hand oriented such that the thumb was facing upwards (except in static position 7) and the elbow facing downwards, and to have the shoulders in a non-elevated position. For position 7, the thumb faced the participant’s mouth in order to simulate the hand position during drinking. At all times, visual cues which showed the current arm configuration as well as the current gesture to be performed, were provided to the participants as text and image on a computer screen (Fig. 3). During the breaks, the participants had time to switch between arm configurations. The experimenter then inspected the arm configuration visually and, if required, provided instructions for adjustment to the participants. The whole process for each arm configuration including all four gestures was repeated five times.

### Signal processing and analysis

There were two possible states of the RHO, i.e., “RHO open” or “RHO closed”. During “RHO open”, only the gestures *RO* or *TC* could be performed, whereas during “RHO closed”, only *RC* and *TO* could be performed. Since the current RHO state is always known and each state effectively only allows two possible gestures, we could use that information to simplify the four-class gesture recognition problem into two separate binary classifications, which in the following will be referred to as “RHO open” (*RO/TC*) and “RHO closed” (*RC/TO*).

In order to detect the user’s intent based from the collected raw FMG signals, different data processing pipelines, i.e., combinations of preprocessing steps and classification algorithms, were examined. Assessed preprocessing steps were: moving average filtering, Min-Max scaling, standard scaling, feature selection, and Principal Component Analysis (PCA), whereas assessed classification algorithms were: Support Vector Machine (SVM), K Nearest Neighbor (KNN), Random Forest (RF), and Linear Discriminant Analysis (LDA) as these have previously shown promising results for gesture classification using FMG [7, 13, 22].

For each participant, out of the five collected repetitions, four were used for classifier training and validation and the remaining repetition was used for testing. Different signal processing pipelines were evaluated in a four-fold leave-one-out-cross-validation manner and the overall highest performing pipeline was then selected to be applied for individual model generation. Reported classification accuracies are test accuracies obtained using the fifth repetition. Additionally, we measured training, as well as inference times. The training time was the time needed to train the model with our training data. The inference time was the time the model needed to make a prediction for a single sample. To measure the latter, we measured the time required for the total test set and divided it by the number of samples in that set.

In order to determine if the data collected in any arm configuration contributed more to the classification performance, a sequential forward selection technique was used [29, 30]. For this technique, we first determined the classification accuracies when only including data from a single configuration into the training set, and the configuration with the highest achieved accuracy was selected. In a second step, data from the selected configuration was combined with data from each of the remaining configurations individually to form the training set with two configurations. The respectively achieved classification accuracies were again compared and the combination achieving the highest accuracy was selected. This procedure was repeated until all configurations were included. The same signal processing pipeline as in the previous analysis was used for all participants. Despite being trained on data collected only in individual arm configurations or combinations thereof, the classifiers were always tested on the full test set which included data from all arm configurations. The resulting classification accuracies were averaged across all participants and both classifications in order to determine the overall contribution of individual arm configurations.

A Wilcoxon signed-rank test at a 0.05 significance level was performed to analyse statistical differences in performance between the classifiers “RHO open” and “RHO closed” as well as for the sequential forward selection of training arm configurations. A Mann–Whitney-U-Test was used to determine statistical differences between the performance of participants using 9 and those using 12 sensors. All data analyses were conducted offline using Python 3.7.12 in Google Colab (12 GB RAM, no use of a GPU).

## Results

### Classification performance

**Fig. 4:**
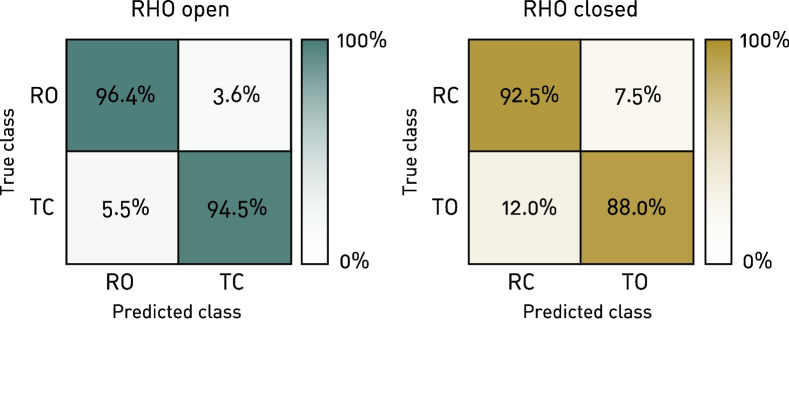
Confusion matrices. Confusion matrices for both classifications, “RHO open” and “RHO closed”, averaged over all arm configurations and all participants, normalized over the ’true’ condition

The pipeline resulting in the highest classification accuracy in validation and which was consequently used for testing included no filtering, no feature selection, standard scaling and a SVM classifier using a Radial Basis Function (RBF) kernel. Parameters were tuned using a grid search. The resulting confusion matrices for each classification averaged over all participants are shown in Fig. 4. For the classification “RHO open”, the true class *RO* was about 1.5 times better predicted than *TC* (error rates $3.6\%$ and $5.5\%$, respectively). Similarly, for the classification “RHO closed”, there were about 1.6 times more false predictions ($12.0\%$) compared with the *RC* class ($7.5\%$).

**Fig. 5:**
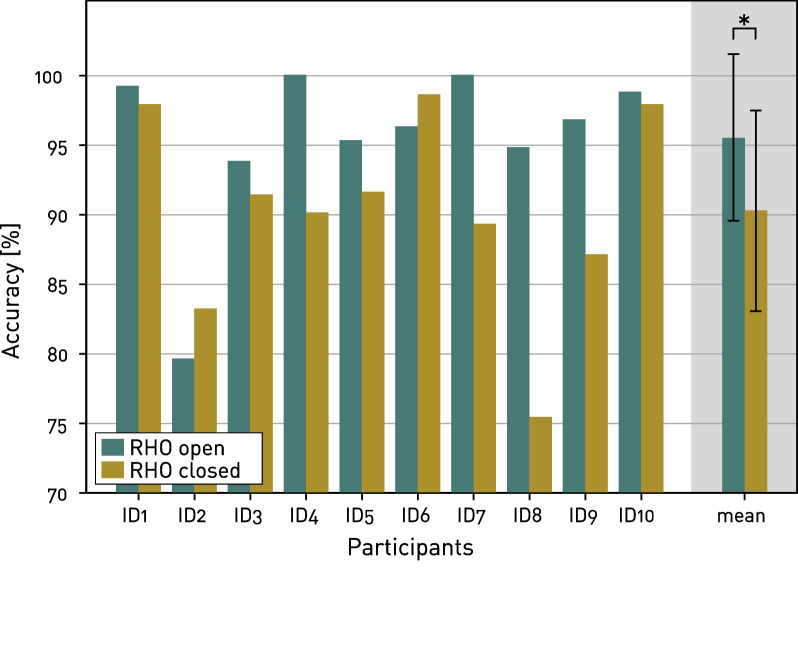
Classification accuracies. Achieved classification accuracies for each individual participant, as well as mean and standard deviation of accuracy across all participant for both classifications “RHO open” and “RHO closed” (*: $p\le 0.05$)

**Fig. 6:**
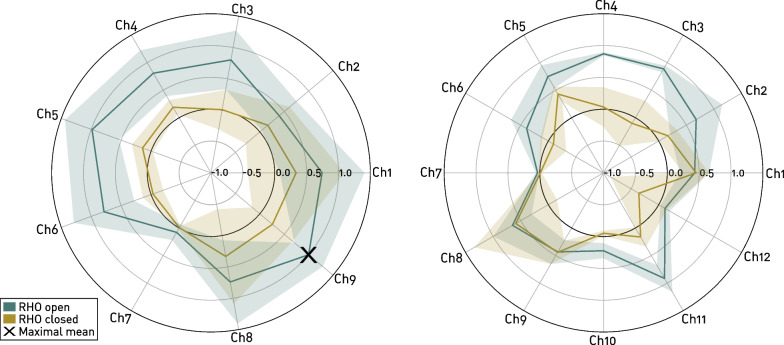
Normalized mean sensor reading differences across all arm configurations. Difference of mean sensor readings (Left: averaged across participants with nine active sensors (n=8), right: averaged across participants with twelve active sensors (n=2)) for both gestures per classification, averaged across all arm configurations for each sensor Ch1-Ch9. Readings are normalized by the overall maximal average difference achieved (indicated by “$\times$”. Bold lines denote averaged difference across participants and arm configurations, transparent areas indicate mean ± standard deviation. Note that sensor readings are not linear and cannot be directly associated with pressure

Individual overall classification accuracies are shown in Fig. 5, ranging between $75.4\%$ (ID8, classifier “RHO closed”) and $100\%$ (ID4 and ID7, classifier “RHO open”). Averaged over both classifiers, participant ID1 achieved the highest accuracy ($98.6\%$) and ID2 performed the worst ($81.4\%$). No significant differences were found between participants using nine (n=8, i.e., ID1, ID3, ID4, ID5, ID6, ID7, ID8, ID9) and those using twelve (n=2, i.e., ID2 and ID10) active sensors ($p > 0.05$). For all participants except ID2 and ID6, the classification “RHO closed” performed worse than “RHO open”, whereas the difference in performance overall was significant ($p = 0.049$). The difference between the distinctness of the gestures in the two classifications is also visible when looking at the difference in raw voltage readings of the sensors (i.e., during “RHO open”, the readings between the gestures *RO* and *TC* were compared and during “RHO closed”, the readings between the gestures *RC* and *TC* were compared). As depicted in Fig. 6, the difference in sensor readings averaged across all nine arm configurations and all participants using nine or twelve active sensors, is smaller for the classification “RHO closed” than “RHO open” for each sensor. However, it has to be noted that the relation from sensor readings to pressure generated by the muscle contraction is not linear.

Classifier training took, on average, 16.35 s (*SD* = 15.06 s) for the classification “RHO open” and 46.44 s (*SD* = 25.20 s) for the classification “RHO closed”, whereas inference times for both classifications were approximately 0.05 ms (*SD* = 0.04ms) and 0.13 ms (*SD* = 0.08 ms) per sample, respectively. The largest measured inference time was 0.26 ms (classification “RHO closed”, ID 9). Significant differences were found in the training and inference times between the two classifications “RHO open” and “RHO closed” (training time: $p = 0.013$, inference time: $p = 0.010$).

### Arm configuration analysis

**Fig. 7:**
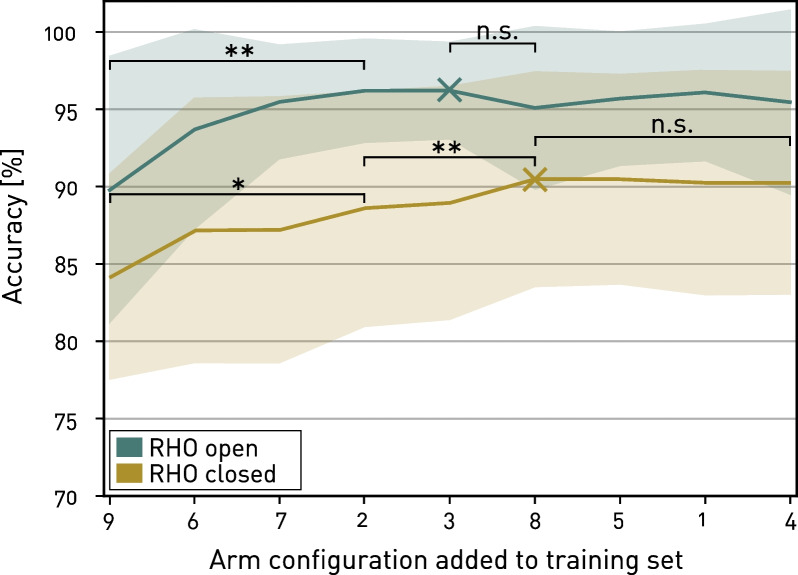
Sequential forward selection of arm configurations. Achieved classification accuracies by adding arm configurations one-by-one during sequential forward selection for both classifications “RHO open” and “RHO closed”. Bold lines denote averaged accuracies across all participants, transparent areas indicate mean ± standard deviation and peaks are indicated by “$\times$” (*: $p\le 0.05$. **: $p\le 0.01$, n.s.: not significant)

The achieved test accuracies during sequential forward selection is given in Fig. 7. The arm configuration which contributed the most to the ability of the model to predict gestures was the dynamic movement 9, i.e., a circular horizontal movement on table level. Listed by the order of contribution, the static positions 6, 7, 2, and 3 followed before the dynamic movement 8 and the three static positions on the right humeral plane, i.e., 5, 1, and 4. For the classification “RHO open”, the peak was achieved after adding position 3, and for the classification “RHO closed” after adding the dynamic movement 8, respectively. Compared to training on position 9 only, the first significant improvement in accuracy for both classifications was found after the three positions 6, 7, and 2 were added ($p_{RHO open} = 0.002$, $p_{RHO closed} = 0.037$). For the classification “RHO open”, no further significant increase was found after adding the remaining positions 3, 8, 5, 1, and 4. However, for the classification “RHO closed”, a further significant improvement was found after additionally adding position 3 and movement 8 ($p = 0.004$).

## Discussion

The aim of this work was to investigate the feasibility of using force myography signals collected from the forearm as an input method to control a robotic hand orthosis worn on the ipsilateral hand. We collected signals from ten participants performing four gestures in nine different arm configurations, i.e., seven static positions and two dynamic movements, corresponding to typical hand use in daily life. We determined corresponding offline classification accuracies and assessed the impact of individual arm configurations in the classifier training data on the overall classifier performance.

### Feasibility of controlling a RHO

The overall achieved average classification accuracy of 92.9% (individual classification *RO/TC*: 95.5%, *RC/TO*: 90.3%) exceeds the target accuracy of 90% proposed by Scheme and Englehart for reasonable, non-frustrating use in upper-limb prosthetics [31]. The total response time of the system, i.e., the time from movement intention to classification, consists of four aspects: the time from movement intention (measurable by electroencephalography) to electrical muscle activation (measurable by EMG), typically around 15–25 ms [32], the time between the onset of electrical muscle activity and the onset of muscle contraction (measurable by FMG), i.e., the electromechanical delay of the muscles, typically around 50 ms [33], the delay by the sampling of the FMG signal, i.e., 10 ms at 100 Hz, as well as the inference time. Thus, even considering the largest measured inference time of 0.26 ms, the total speed of response adds up to only approximately 85 ms, which is still more than fast enough to produce predictions in a real-time application, assuming that the bandwidth of human hand movement is typically below 4.5 Hz [34]. We can assume that this holds true even when switching to a microcontroller on-board the RHO with less processing power than the processor used for the analysis in this study, since the inference time makes only a very small fraction of the overall response time. Accordingly, classification accuracy and response time indicate general feasibility, but the fact that one of the classification accuracies only marginally falls above that limit calls for further investigation.

FMG has previously been examined for the control of an RHO by Yap et al. [15] who reported 95% online classification accuracy distinguishing four hand gestures in a fixed elbow configuration in three neurologically-intact participants in a setting where FMG was measured from the forearm contralateral to the RHO. Such a contralateral setting could foster bilateral movement training, which in turn can promote functional recovery in the contralateral paretic hand [35]. However, for practical control of an assistive RHO in activities of daily living, it is important that FMG data is acquired on the forearm ipsilateral to a worn RHO so that it does not limit bimanual activities. No previous studies have reported quantitative performance metrics such as classification accuracies using FMG in the ipsilateral setting. Xiao et al. [17] employed the ipsilateral setting in their study to help the participants familiarize themselves with the control of a RHO by using FMG signals collected from their ipsilateral forearm. Esposito et al. [18] implemented both, contra- and ipsilateral control and selected between those settings depending on the quality of the measurable FMG signal of the user. However, neither of these studies reported any quantitative results of the ipsilateral setting.

Despite only conducting a presumably simple binary classification compared to previous works distinguishing more gestures, the classification accuracies achieved in our work were not notably superior to other studies that employed FMG for gesture recognition. Using a similar sensor setup as the one used for our work, Xiao et al. [19] detected grasping actions in pick and place tasks with a comparable accuracy of 92% in neurologically-intact participants; Jiang et al. [29] were able to distinguish between 48 hand gestures in a cross-trial evaluation with an accuracy of 83.5%. The main reason that we could not achieve notably higher accuracies than these studies is likely the inherent physical restrictions posed by the RHO on the hand. Therefore, the gestures to classify were not as distinguishable as, e.g., a fully open hand and a closed fist, but rather resembled an isometric muscle contraction in the transition phases between the current states of the RHO (trying to close while RHO open/trying to open while RHO closed). In addition, Xiao et al. [19] also reported significantly higher prediction accuracies when using FMG signals collected from the wrist instead of the forearm. However, for many RHO, the wrist is covered by the device and does therefore not allow placement of FMG sensors in that area. All these observations support that wearing the RHO makes it more difficult to get distinguishable signals and thus correctly classify a desired action compared to gesture classification without wearing a RHO.

The significantly worse performance of the classification “RHO closed” compared to “RHO open” matched the expectations from observations during data collection and unstructured feedback by the participants. In neurologically-intact participants, finger flexors are usually stronger than finger extensors [36]. This leads to the expectation that during flexion (i.e., gesture *TC*) larger and hence more distinguishable FMG signals could be measurable than during extension (i.e., gesture *TO*). Further, when the RHO was open, the participants only had to counteract the stiffness of the springs in the fingers to perform the gesture *TC*. However, when the RHO was closed, it provided an additional force intended for grasp assistance, which the participants had to counteract in order to perform the gesture *TO*. Thus, it might be that the maximum force applied by the participants, as instructed by the experimenters, was too small to achieve a more distinct volumetric change in the muscles.

Looking at the different error modes in Fig. 4, we can distinguish between two types of failure for each classification: when a desired change of RHO-state is wrongly detected or when an actual desired change is not detected. The most frequently occurring error was found when the participants tried to change the state from RHO closed to open (true class *TO*), yet the RHO stayed closed (predicted class *RC*). While this type of failure might be annoying, it can usually be solved by just trying to conduct the desired gesture again. On the other hand, a misclassification when intending to keep the RHO closed yields an unintended opening (true class *RC*, predicted class *TO*). When the user is holding an object, this leads to dropping it. This failure mode is therefore considered to be the most critical. Although this failure mode occurred less than $7\%$ of the time, it could be considered to adapt the decision threshold of the classifier for future iterations, in order to reduce the risk even further at the expense of a potential increase of undetected opening attempts [37].

### Variation in performance between participants

Some variation in performance between participants was observed. Out of the ten participants, only six achieved an acceptable classification accuracy above $90\%$ for both classifications “RHO open” and “RHO closed”. Further, although the classification “RHO open” performing overall significantly better than “RHO closed”, there were two participants for which the contrary was observed. Besides the participant’s individual ability to generate consistent muscle activations, further sources potentially introducing variability could be differences in band tightness based on the participant’s oral feedback [7], inconsistency in the sensor locations as previously investigated in EMG [38], e.g., after prolonged wearing time, or the amount of force applied against the finger mechanism of the RHO to perform the intended gesture.

An approach to try to compensate for the observed inter-participant variability is to use individually optimized data processing pipelines, i.e., combinations of preprocessing steps and classification algorithms, instead of a general pipeline for all participants. However, when investigating this option in a preliminary analysis, we found that such individual pipelines only performed marginally better in validation and, when translating to unseen data during testing, they performed even slightly worse than the general pipeline which was used in this work. Using the same pipeline across all participants leads to simpler processing and is consistent with other studies involving neurologically-intact participants [15, 19, 29]. However, it should be investigated whether an individual pipeline could provide a meaningful improvement in accuracy in case of lower muscle activations, e.g., for users with sensorimotor hand impairments.

### Arm configuration analysis

Analysing the contribution of individual arm configurations to the overall classification performance, we found that the dynamic movement 9, i.e., the one horizontally on table level, contributed the highest. Including dynamic movements provides data with a higher variability in the training set, which in turn can make a model more suitable for testing in scenarios which also include such variabilities, such as eating or other activities of daily living. The three highest contributing static positions 6, 7, and 2 required the participants to extend the elbow straight to the left humeral plane (Positions 6 and 2) or to mouth level on the sagittal plane close to the body with a flexed elbow. Including these three static positions on different vertical levels yielded in a significant improvement in classification accuracy compared to only using one dynamic movement on table level, matching the suggestion by Radmand et al. [27] to include positions in both, straight and bent elbow configurations. Introducing the second dynamic movement, 8, resulted in a further significant increase for classification “RHO closed”. This observation matches our expectation, since adding this movement not only introduced dynamic, and thus highly variable data, but also, for the first time, introduces data from the right humeral plane. Accordingly, the data from static positions 5, 1 and 4, which all lie in the same plane as movement 8 might be partially redundant and therefore don’t contribute to further significant improvements. However, surprisingly, introducing the dynamic movement 8 led to a decrease in accuracy for classification “RHO open”. Yet, as the decrease was not significant and the achieved accuracy is still relatively high ($>95\%$) we assume that in this case the additionally introduced variability in data was not required since a plateau was already achieved earlier.

For this work, no data collection time constraints (i.e., large number of repetitions and arm configurations) were considered for the sake of achieving a large training set for the investigations. However, in real-life applications, such a long training time is critical as the users may lose motivation and experience fatigue during prolonged training. These findings lead to the conclusion that the static positions 4, 1, and 5 (in that order) could be removed in a future training data collection without notably decreasing the classification accuracy, reducing the overall training time and therefore the burden on the participants.

### Limitations and future work

While this study provided valuable novel insights and a first indication towards the feasibility of using FMG to control a RHO, important aspects need to be taken into account when transferring these findings to practical applications. In a first step, the feasibility should also be investigated for online control. Previous works using EMG have found significantly worse performance in online classification compared to offline [39]. Further, all study participants were neurologically-intact, male, and represented a relatively low variability in age and forearm circumference. Unfortunately, there is only very limited data available on FMG signals collected from people with neurological hand impairments. For grasping detection of one grasp type, Sadarangani et al. [40] found that, compared to neurologically-intact individuals, individuals after stroke achieved inferior, but still acceptable ($>90\%$) classification accuracies. For people with hand impairments due to spinal cord injury, no such data is available. However, in this population, the residual muscle activity (and therefore the volumetric change) in the forearm depends on the type and level of lesion which could impact the distinctness of measurable FMG signals. For both these reasons, we expect that the classification accuracy in online control for people with neurological hand impairment would be lower than what was reported in this study. In order to improve online classification accuracy to be suitable for this population, some improvements on the hardware and the data collection are needed. On the hardware side, fusing data from multiple sensor types could be considered. Previous works have shown superior performance when using EMG and FMG data simultaneously compared to only EMG or only FMG [13, 41]. Further, the inclusion of inertial measurement units could identify dynamic motions in order to avoid misclassifications when transitioning between different hand positions during activities of daily living [42]. From an application point of view, different gestures could be used to trigger the desired actions. While the gestures used in this study were selected to resemble the targeted action (e.g., “trying to close” in order to close the hand), other less intuitive gestures such as, e.g., a simple co-contraction of the forearm, might be easier to perform for the participants and produce better distinguishable FMG signals [43]. Further, with data from a larger number of participants, more sophisticated signal analysis such as transfer learning could further allow for improved classification accuracies while keeping the required amount of training in an acceptable range [44].

### Conclusions

This work, for the first time, assessed the feasibility of classifying opening and closing based on FMG data from the forearm while wearing a robotic hand orthosis on the ipsilateral hand in neurologically-intact participants. In an offline analysis, we found that using FMG could be a viable intention detection strategy for such assistive devices, yet for a more conclusive statement, further investigations involving people with hand impairments are required. Additionally, this work identified trade-offs between gesture recognition accuracy and the burden on the user during collection of training data and determined methods to optimize the training procedure and time without reducing gesture classification performance. Based on these findings, methods were identified which could potentially overcome challenges arising when transferring such technologies to their intended context of use, i.e., assisting people with sensorimotor hand impairments during activities of daily living.

## Data Availability

The dataset supporting the conclusions of this article is available in the ETH Zurich Research Collection repository, DOI: 10.3929/ethz-b-000596647.

## Abbreviations

EMG: Electromyography
FMG: Force myography
FSR: Force-sensitive resistors
RHO: Robotic hand orthosis
RO: Relaxed open (gesture)
TC: Trying to close (gesture)
RC: Relaxed closed (gesture)
TO: Trying to open (gesture)
PCA: Principal Component Analysis
SVM: Support Vector Machine
KNN: K Nearest Neighbor
RBF: Radial Basis Function
RF: Random Forest
LDA: Linear Discriminant Analysis
